# Genetically and functionally defined NTS to PBN brain circuits mediating anorexia

**DOI:** 10.1038/ncomms11905

**Published:** 2016-06-15

**Authors:** Carolyn W. Roman, Victor A. Derkach, Richard D. Palmiter

**Affiliations:** 1 Department of Biochemistry, University of Washington, Seattle, Washington 98195, USA; 2 Howard Hughes Medical Institute, University of Washington, Seattle, Washington 98195, USA

## Abstract

The central nervous system controls food consumption to maintain metabolic homoeostasis. In response to a meal, visceral signals from the gut activate neurons in the nucleus of the solitary tract (NTS) via the vagus nerve. These NTS neurons then excite brain regions known to mediate feeding behaviour, such as the lateral parabrachial nucleus (PBN). We previously described a neural circuit for appetite suppression involving calcitonin gene-related protein (CGRP)-expressing PBN (CGRP^PBN^) neurons; however, the molecular identity of the inputs to these neurons was not established. Here we identify cholecystokinin (CCK) and noradrenergic, dopamine β-hydroxylase (DBH)-expressing NTS neurons as two separate populations that directly excite CGRP^PBN^ neurons. When these NTS neurons are activated using optogenetic or chemogenetic methods, food intake decreases and with chronic stimulation mice lose body weight. Our optogenetic results reveal that CCK and DBH neurons in the NTS directly engage CGRP^PBN^ neurons to promote anorexia.

## Abstract (web summary)

Neurons in the nucleus of the solitary tract (NTS) are known to receive visceral signals from the gut during feeding. Here, the authors identify two populations of CCK- and DBH-expressing NTS neurons that work to suppress food intake when activated via opto- or chemogenetic stimulation.

## Graphical abstract

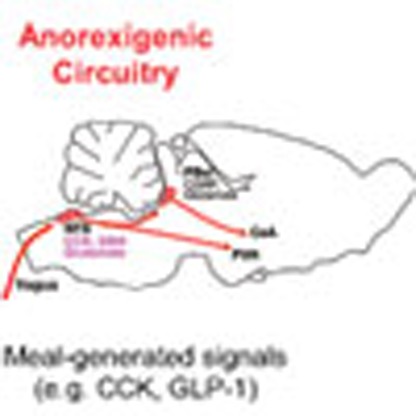

Metabolic homoeostasis requires coordination of central circuits that balance energy input with its output. Coordination is achieved via long-term signals responding to energy status as well as short-term signals, such as those that drive the initiation or termination of a meal. Following ingestion, gut distention and satiety hormones released from the intestine and pancreas are detected by vagal afferents that relay this information to the area postrema and nucleus of the solitary tract (NTS) neurons in the brainstem^1^. The relevant, satiety-related neurons in the NTS receiving this information relay it to other nuclei involved in suppressing feeding behaviour. One of the major targets receiving viscerosensory information from the NTS is the lateral parabrachial nucleus (PBN)^2^,^3^,^4^,^5^,^6^.

Our lab recently described a neural circuit mediating appetite suppression that involves a distinct population of neurons in the lateral PBN expressing the neuropeptide CGRP (CGRP^PBN^ neurons)^7^. Activation of CGRP^PBN^ neurons or their projections in the central nucleus of the amygdala (CeA) decreases feeding by hungry mice. Consistently, inhibition of CGRP^PBN^ neurons attenuates anorexic responses to satiety hormones, and other anorexigenic agents such as lithium chloride and lipopolysaccharide^7^. Furthermore, this population of neurons and their excitatory inputs from the NTS are necessary for the starvation phenotype that occurs in mice following ablation of hypothalamic agouti-related peptide (AgRP)-expressing neurons^6^,^7^. Either inhibition of CGRP^PBN^ neurons or inactivation of glutamatergic signalling from the NTS to the PBN can rescue these mice from starvation after AgRP neuron ablation^6^,^7^. The excitatory input driving CGRP neurons is, therefore, an important component of the functionally identified CGRP^PBN^-to-CeA anorexic neural circuit. In this study, we aimed to identify NTS neurons with direct excitatory connections to CGRP^PBN^ neurons and explore their role in feeding behaviour.

The NTS is a heterogeneous brain region with many neuronal types expressing a variety of neuropeptides and neuromodulators^8^,^9^,^10^. We applied contemporary neuron-manipulation techniques to two distinct populations of NTS neurons (cholecystokinin (CCK)- and dopamine β-hydroxylase (DBH)-expressing neurons) that have been implicated in feeding behaviour. CCK- and DBH-expressing neurons are located in the caudal portion of the NTS, which is widely accepted as the ‘visceral' portion of the NTS, receiving input from the gut via the vagus nerve, in contrast to the rostral portion, which receives and relays taste information in the rodent^11^,^12^. CCK is well-known for its ability to decrease feeding. CCK is a satiety hormone produced and released by cells in the gastrointestinal tract in response to food intake and acts on a number of systems to signal the presence of food and ultimately contribute to meal termination^13^. However, CCK is also a neuropeptide and it can suppress feeding when it is administered centrally^14^,^15^. There is relatively little information regarding the function of CCK-expressing neurons within the caudal NTS; however, anatomical studies demonstrated that these neurons project to the lateral PBN^3^, the area where anorexigenic CGRP^PBN^ neurons reside. While norepinephrine has been shown to both increase and decrease feeding, depending on the site and type of receptors activated (for review see ref. 16), many studies implicate the A2 population of DBH-expressing cells in the NTS in satiety. For example, A2 neurons are activated by the satiety hormone CCK^17^ as well as by large meals in rats^18^. Furthermore, anorexia induced by either CCK or lithium chloride is significantly attenuated in rats in which A2 noradrenergic neurons in the NTS are ablated using a saporin toxin-conjugated DBH antibody^19^,^20^. In addition, anatomical studies establish a dense projection from DBH neurons in the caudal NTS to the lateral PBN^4^,^21^.

Therefore, we hypothesized that CCK^NTS^ and DBH^NTS^ neuronal projections to CGRP^PBN^ neurons might mediate anorexia. First, we provide evidence that these two separate populations of NTS neurons become activated during feeding and not during a fast. We demonstrate that artificial activation of either CCK^NTS^ or DBH^NTS^ neurons, or their axonal projections within the PBN, is sufficient to attenuate food intake by hungry mice. We then use channelrhodopsin-assisted analysis to demonstrate that both CCK^NTS^ and DBH^NTS^ neurons engage CGRP^PBN^ neurons via direct glutamatergic inputs.

## Results

### Food intake activates genetically distinct NTS neurons

**Figure 1:**
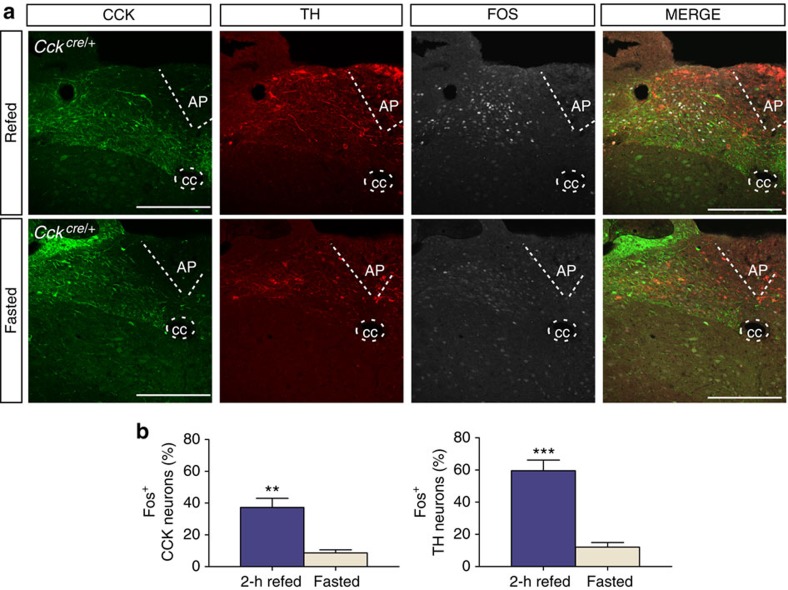
CCK^NTS^ and DBH^NTS^ neurons are activated by food intake. (**a**) Representative NTS images showing, CCK-, TH- and Fos-immunoreactivity in a refed animal (top panels) and a fasted animal (bottom panels). Scale bar, 100 μm. (**b**) Quantification of Fos labelling in CCK- and TH-immunoreactive cells (unpaired *t*-test). Data represent mean (*n*=5 refed, 4 fasted)±s.e.m., **P<0.01, ***P<0.001. AP, area postrema; cc, central canal.

To substantiate a role for both CCK^NTS^ and DBH^NTS^ neurons in food intake, we compared activation of these cells in animals that had been fasted to those that had fasted and then fed. To visualize CCK^NTS^ neurons, a Cre recombinase-dependent adeno-associated virus (AAV) virus (AAV1-DIO-YFP) that allows expression of yellow fluorescent protein (YFP) was injected into the NTS of *Cck*^*Cre/+*^ mice. To label DBH^NTS^ neurons, we used an antibody to tyrosine hydroxylase, an essential enzyme for norepinephrine biosynthesis. Mice were fasted overnight after which one group of mice (refed group) was allowed unrestricted access to normal chow for 2 h. The total number of CCK and DBH neurons per slice was the same between groups; however, the refed group showed significantly greater amount of Fos immunoreactivity in both the YFP- and tyrosine hydroxylase-positive neurons compared with the fasted group (Fig. 1a,b). Approximately 40 and 60% of the CCK- and DBH-expressing neurons expressed Fos, respectively. These data corroborate previous reports showing that DBH^NTS^ neurons are activated by a large meal^18^, and demonstrate that CCK^NTS^ neurons are also activated by food intake.

Analysis of CCK^NTS^ and DBH^NTS^ neurons within the same coronal brain sections across several levels of the mid-caudal NTS clearly revealed that they are two distinct populations of neurons. Brain slices (average of 3 each) from 17 individual *Cck*^*Cre/+*^ mice injected into the NTS with AAV1-DIO-YFP were labelled using a tyrosine hydroxylase antibody. No overlap between CCK-expressing cells and tyrosine hydroxylase-positive cells was observed at any level of the NTS, except for a single neuron in one animal at the level of the area postrema. This separation agrees with studies in the rat showing that A2, noradrenergic neurons do not co-express CCK, in contrast to more rostral C2, adrenergic neurons^22^,^23^. CCK^NTS^ and DBH^NTS^ populations were observed at similar expression levels across the NTS and were intermixed throughout, although CCK neurons appeared at slightly more lateral portions within the NTS than DBH neurons (Supplementary Fig. 1).

### DREADD activation increases firing rate of NTS neurons

**Figure 2:**
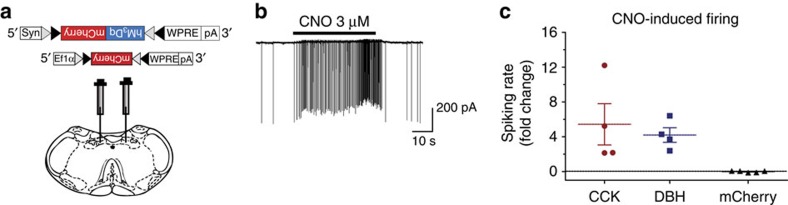
Activation of NTS neurons via a Gq-coupled receptor increases firing rate *in vitro*. (**a**) Diagram depicting viral targeting site in a coronal section through the medial NTS of the mouse brainstem. (**b**) Example of spiking activity recorded from a CCK^NTS^ neuron expressing hM3Dq:mCherry before and after addition of 3 μM CNO. (**c**) Application of 3 μM CNO increases firing rate in CCK^NTS^ and DBH^NTS^ cells expressing hM3Dq:mCherry (*n*=4 cells from two mice each group). No change is observed in cells expressing mCherry only (*n*=5 DBH^NTS^ cells from one mouse). Effect is measured as fold change in spiking frequency from its basal level before CNO, error bars represent s.e.m.

Selective activation and visualization of CCK^NTS^ and DBH^NTS^ neurons was achieved by NTS injection of AAV encoding a Cre-dependent Gαq-coupled designer receptor exclusively activated by designer drugs (DREADD) fused to the fluorescent protein mCherry (AAV1-DIO-hM3Dq:mCherry). Intraperitoneal (i.p.) injection of the otherwise inert ligand, clozapine-N-oxide (CNO), activates the hM3Dq receptor *in vivo*. This technique has been shown to increase activity in several different neuronal types^24^,^25^. To determine whether CNO could directly activate CCK^NTS^ and DBH^NTS^ neurons expressing the hM3Dq receptor, electrophysiological analysis was performed on NTS brain slices taken from *Cck*^*Cre/+*^ and *Dbh*^*Cre/+*^ mice that had been injected at least 3 weeks before with either AAV1-DIO-hM3Dq:mCherry (hM3Dq) or, as a control, AAV1-DIO-mCherry (Fig. 2a). Spiking was recorded using cell-attached patches. In the presence of CNO, both CCK^NTS^ and DBH^NTS^ neurons reliably (4/4 in both cases) increased the frequency of action potentials several fold compared with the vehicle (Fig. 2b,c). In contrast, CNO had no effect on cells expressing mCherry alone (Fig. 2c). The increase was observed in the presence of glutamate and GABA receptor inhibitors, suggesting a direct effect of CNO on these cells.

### Activation of CCK^NTS^ or DBH^NTS^ neurons decreases feeding

**Figure 3:**
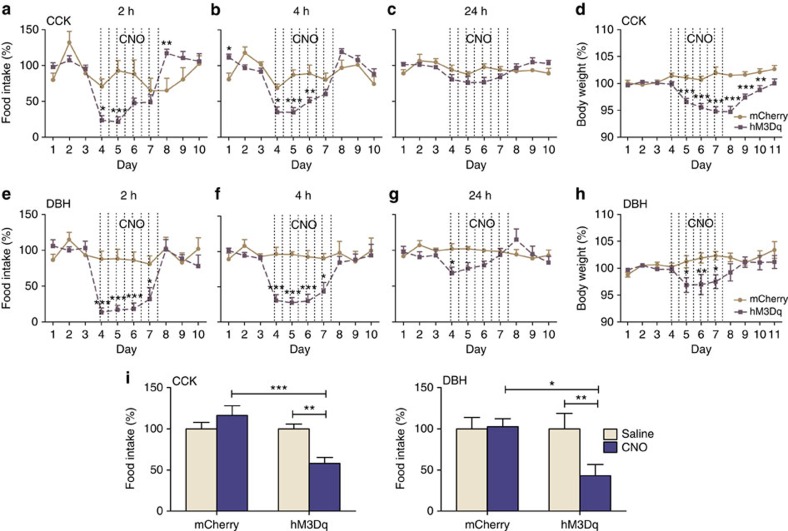
Activation of CCK^NTS^ or DBH^NTS^ neurons suppresses food intake. (**a**–**c**) Chemogenetic activation of CCK^NTS^ neurons decreases baseline food intake at 2 h (**a**) and 4 h (**b**) after lights out, but has no significant effect on 24-h food intake (**c**). (**e**–**g**) Chemogenetic activation of DBH^NTS^ neurons decreases baseline food intake at 2h (**e**) and 4 h (**f**) after lights out during all 4 days of CNO injection, but only effects 24-h food intake (**g**) on the first day of CNO injection. (**d**,**h**) Activation of CCK^NTS^ (**d**) or DBH^NTS^ (**h**) neurons decreases body weight. (**i**), In food restricted animals, activation of CCK^NTS^ or DBH^NTS^ neurons decreases food consumed over 1 h during the day compared to saline injection or CNO injection into mice expressing only mCherry. Data were analysed using two-way repeated measures ANOVA, bonferroni *post hoc* comparison, and represent mean (CCK: *n*=6 mCherry, 9 hM3Dq, DBH: *n*=6 per group)±s.e.m., *P<0.05, **P<0.01, ***P<0.001.

To investigate the effect of CCK^NTS^ neuronal activation on food intake, we bilaterally transduced neurons in the NTS of *Cck*^*Cre/+*^mice with either the excitatory hM3Dq receptor or, as a control, mCherry. Saline was injected i.p. twice daily for 3 days to establish baseline food intake. During baseline measurements, no differences in cumulative amount of food intake were observed between the two groups of mice at 2, 4 or 24 h after lights out (data not shown). Subsequently, CNO was injected (0.8 mg per kg body weight i.p.) twice daily over the course of 4 days. Treatment with CNO decreased feeding in hM3Dq-expressing animals by >50% compared with baseline when measured at 2 and 4 h after lights out (Fig. 3a,b); however, 24-h food intake was not significantly influenced (Fig. 3c). The same experimental paradigm was carried out using *Dbh*^*Cre/+*^ mice. We observed significantly decreased food intake at both the 2- and 4-h time points after lights out (Fig. 3e,f), whereas 24-h food intake was only affected on the first day after CNO injection (Fig. 3g). Meal analysis revealed that the decrease in food intake was due to both a decrease in meal size and meal number for both genotypes (Supplementary Fig. 2).

Compared with CNO-treated controls, both *Cck*^*Cre/+*^ and *Dbh*^*Cre/+*^mice transduced with the virus expressing hM3Dq decreased their body weight over the course of the experiment (Fig. 3d,h). Although moderate, the significant drop in body weight without consistent changes in 24-h food intake suggests that activation of CCK^NTS^ or DBH^NTS^ neurons also increases energy expenditure.

In addition to reducing food intake during the normal feeding cycle, we also observed that activation of CCK^NTS^ or DBH^NTS^ neurons with hM3Dq and CNO is sufficient to suppress food intake by hungry mice. For this experiment, mice were food restricted and kept at 85–90% of their pre-restriction body weight. CNO or saline was injected during the light cycle, 45 min later food was provided and the amount consumed in 1 h was measured. Activation of CCK^NTS^ neurons with CNO decreased 1-h food intake by 42%, while activation of DBH^NTS^ neurons decreased food intake by 57% when compared with intake after saline injection (Fig. 3i). No differences were observed between saline and CNO treatment in animals expressing mCherry only.

### Activation does not induce anxiety-like behaviour

Because behavioural state can influence feeding, we examined whether the inhibition of food intake was due to anxiety. Mice from the previous experiments were placed in an open-field arena 45 min after injection of CNO. No differences were observed between mCherry- and hM3Dq-expressing mice in the time spent in the centre of the open field providing evidence that activation of CCK^NTS^ or DBH^NTS^ neurons does not cause overt anxiety (Supplementary Fig. 3a). Although there was a significant decrease in total distance travelled after CNO injection in mice expressing hM3Dq in DBH^NTS^ neurons and a trend towards a decrease in the CCK group (Supplementary Fig. 3b), this result is likely due to the satiation effect of activating these neurons because mice that are not hungry forage less in a novel environment.

### Activation of CCK^NTS^ or DBH^NTS^ neurons induces Fos

**Figure 4:**
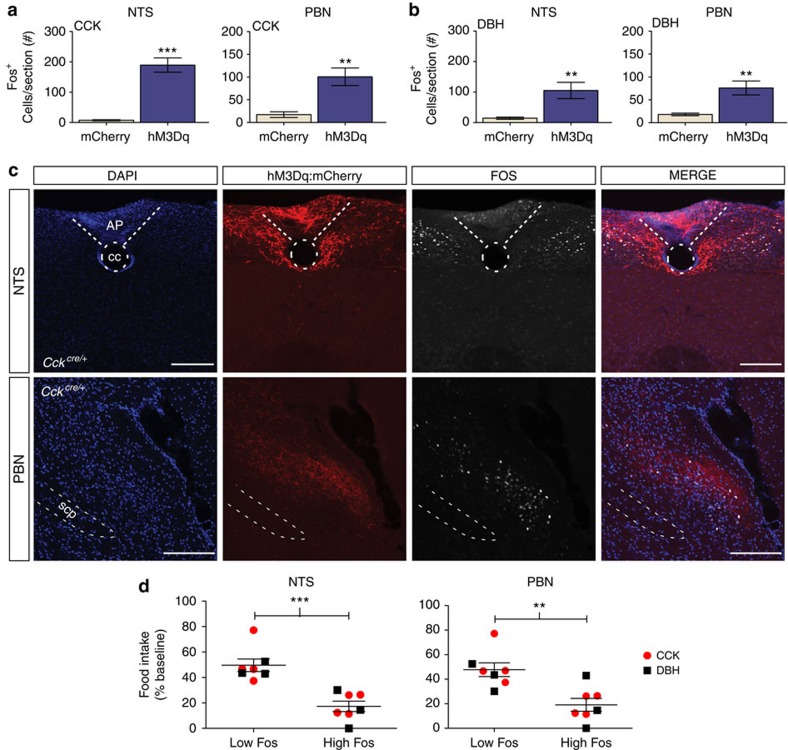
CCK^NTS^ or DBH^NTS^ neuronal activation induces Fos in the NTS and in the PBN. (**a**,**b**) Compared with animals expressing only mCherry transgene (*n*=6 for each genotype), *Cck*^*Cre/+*^ (**a**) and *Dbh*^*Cre/+*^ (**b**) mice expressing hM3Dq:mCherry in the NTS show increases in Fos expression in the NTS and PBN after CNO injection (unpaired *t*-test). Data represent mean (CCK: *n*=8 PBN, 9 NTS, DBH: *n*=6)±s.e.m. (**c**) Representative immunohistological sections demonstrating hM3Dq:mCherry and Fos expression in the NTS (top panels) and the PBN (bottom panels) after CNO injection. Scale bar, 100 μm. (**d**) Quantification of post-CNO 4-h food intake comparing individual hM3Dq low- and high-Fos-expressing animals for both the NTS and PBN (unpaired *t*-test). Data from *Cck*^*Cre/+*^ mice are represented by red circles, *Dbh*^*Cre/+*^ mice by black squares, lines represent mean±s.e.m., **P<0.01, ***P<0.001. AP, area postrema; cc, central canal; scp, superior cerebellar peduncle.

One week after mice underwent food intake and behavioural experiments, CNO was injected and brain tissue was evaluated 3 h later for Fos immunoreactivity as an indirect marker of neuronal activity. Animals expressing hM3Dq showed abundant Fos immunoreactivity in the NTS and the lateral PBN (Fig. 4a–c). In contrast, minimal Fos labelling was observed in these regions in mice expressing mCherry alone. Importantly, the amount of Fos labelling observed in both the NTS and PBN was associated with the degree of CNO-induced suppression of feeding in both *Cck*^*Cre/+*^ and *Dbh*^*Cre/+*^mice expressing hM3Dq. For this analysis, each genotype was split into a high- and a low-Fos-expressing group based on the median. Average food intake for the first 4 h after CNO injection was then compared for each group. In both the NTS and the PBN, the high-Fos group had significantly greater suppression of food intake when compared with the low-Fos group (Fig. 4d). For simplicity of presentation, the CCK^NTS^ and DBH^NTS^ groups were combined; however, the results are also significant when analysed separately.

Within the PBN, a substantial portion of Fos labelling following CNO activation of either CCK^NTS^ or DBH^NTS^ neurons co-localized with CGRP immunoreactive neurons (Supplementary Fig. 4). The distribution of co-labelled cells was similar anatomically to that observed after refeeding after a fast^26^. In both studies, a subset of CGRP^PBN^ neurons that were Fos-negative was detected in the most ventral lateral aspect of the external lateral PBN within an area of the PBN with a dense clustering of CGRP-immunopositive fibres.

Mice expressing the hM3Dq receptor displayed CNO-induced activation in a number of discrete brain regions, which may reflect direct or indirect projections from CCK^NTS^ or DBH^NTS^ neurons. In addition to the NTS and the PBN, increased Fos immunoreactivity was encountered in several forebrain regions including the bed nucleus of the stria terminalis (BNST), amygdala and paraventricular nucleus of the hypothalamus (Supplementary Fig. 5a–c). Within the BNST, the vast majority of expression was restricted to the oval nucleus (BNSTov). Within the amygdala, dense expression occurred almost exclusively within the CeA. Sparse Fos expression was also observed in the periaqueductal grey (Supplementary Fig. 5d). Minimal Fos labelling was observed in these regions in control mice expressing mCherry alone.

Injection of CNO activated CCK^NTS^ neurons that were virally transduced to express hM3Dq. However, when we analysed Fos in the NTS following CNO injection, the majority of Fos-positive NTS cells did not express hM3Dq:mCherry, suggesting that non-CCK neurons were also activated following CNO. We found ∼20% of the noradrenergic cells in the NTS co-expressed Fos (Supplementary Fig. 6). In addition, a dense network of local mCherry-expressing fibres was observed in the NTS of *Cck*^*Cre/+*^ animals expressing hM3Dq:mCherry or mCherry. This finding suggests that activation of CCK^NTS^ neurons stimulates DBH^NTS^ neurons and other unidentified neurons in the NTS. Activation of DBH^NTS^ neurons also appeared to induce Fos in non-DBH neurons; however, this effect was almost entirely restricted to more rostral portions of the NTS. In the caudal NTS, DBH^NTS^ neuronal fibres appeared more restricted, unlike the extensive web-like network of CCK^NTS^ neuron fibres in the same area.

### CCK^NTS^ and DBH^NTS^ neurons directly activate CGRP^PBN^ neurons

**Figure 5:**
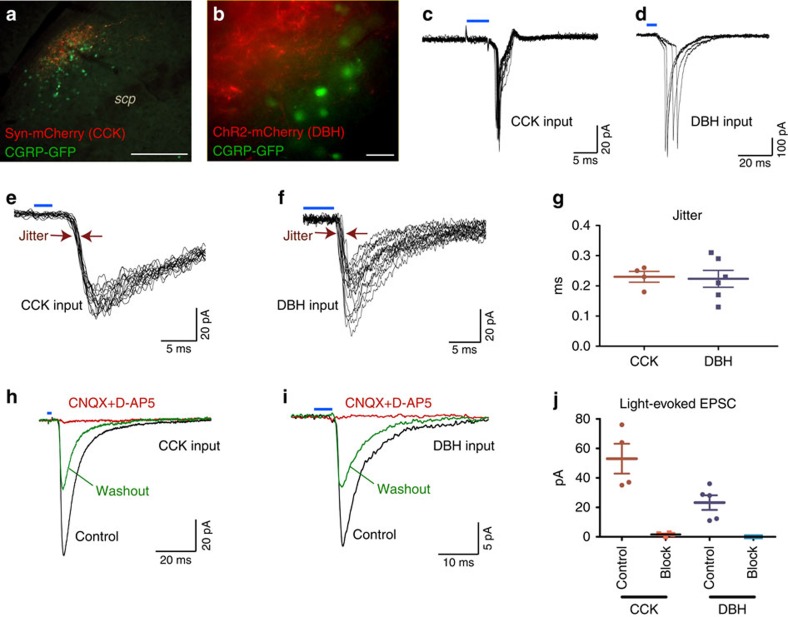
CCK^NTS^ and DBH^NTS^ neurons send direct, excitatory input to CGRP^PBN^ neurons. (**a**) Representative immunohistological image from the PBN showing CCK fibre terminals in red and CGRP-expressing cell nuclei in green after injecting AAV1-DIO-synaptophysin:mCherry into the NTS of a *Cck*^*Cre/+*^*::Calca*^*Cre:GFP/+*^ mouse. Scale bar, 100 μm. (**b**) Live imaging of the lateral PBN before electrophysiological recording in a *Dbh*^*Cre/+*^*::Calca*^*Cre:GFP/+*^ mouse. Red fibres, from DBH^NTS^ neurons targeted for photostimulation and green nuclei indicate CGRP^PBN^ neurons targeted for recording. Scale bar 20 μm. (**c**,**d**) Action potentials recorded in cell-attached configuration and evoked in CGRP^PBN^ neurons by stimulation of CCK^NTS^ (**c**) and DBH^NTS^ (**d**) axons by pulses of blue light indicated by blue bars. (**e**,**f**) Synaptic currents in CGRP^PBN^ neurons following stimulation of CCK^NTS^ (**e**) and DBH^NTS^ (**f**) inputs by pulses of blue light indicated by blue bars. (**g**) Individual jitter values for synaptic currents in CGRP^PBN^ neurons represented in **e** and **f**. (**h**,**i**) Representative traces from synaptic currents in CGRP^PBN^ neurons, following stimulation of CCK^NTS^ (**h**) and DBH^NTS^ (**i**) inputs were reversibly inhibited by 20 μM CNQX and 100 μM D-AP5. (**j**) Quantitative effect of 20 μM CNQX and 100 μM D-AP5 (block) on synaptic currents represented in **h**,**i**. All currents were recorded in the presence of 100 μM picrotoxin.

Because CNO-mediated activation of CCK^NTS^ or DBH^NTS^ neurons induced Fos expression in a sub-population of CGRP^PBN^ neurons, we examined whether there are direct synaptic connections between the genetically defined NTS populations and CGRP^PBN^ neurons. For these experiments, double knock-in *Cck*^*Cre/+*^*::Calca*^*Cre:GFP/+*^ or *Dbh*^*Cre/+*^*::Calca*^*Cre:GFP/+*^ mice were generated to allow visualization of the nuclear-localized Cre:GFP from the *Calca* locus that encodes CGRP. These mice were injected into the NTS with AAV1-DIO-synaptophysin:mCherry virus. This allowed us to visualize the close proximity of mCherry-labelled CCK^NTS^ or DBH^NTS^ axons with GFP-expressing, CGRP neurons in the lateral PBN (Fig. 5a). The mCherry-labelled axons of CCK^NTS^ or DBH^NTS^ neurons in the PBN were predominantly lateral to the CGRP^PBN^ cell bodies where there is an extensive dendritic field^27^,^28^,^29^.

For electrophysiological recordings, PBN slices were obtained from double knock-in mice previously injected into the NTS with Cre-dependent AAV1-DIO-ChR2:mCherry that expresses channelrhodopsin:mCherrry fusion protein. This preparation allowed excitation within the lateral PBN of genetically identified, mCherry-labelled fibres from CCK^NTS^ or DBH^NTS^ neurons while recording responses from GFP-labelled CGRP neurons (Fig. 5b). Because of the dense population of CGRP neurons that reside in the hypoglossal nucleus near the NTS, we first performed control experiments and verified that injections into the NTS and hypoglossal nucleus of a *Calca*^*Cre:GFP/+*^ mouse did not label any projections to the PBN. Recording the activity of GFP-labelled CGRP^PBN^ neurons in slices from these mice revealed that they are intrinsically active with a low firing frequency (2.4±0.45 Hz, *n*=23), resting membrane potential of −71.3±1.1 mV and a high input resistance (1.12±0.11 GΩ, *n*=23). Photoactivation of nearby ChR2:mCherry fibres from CCK^NTS^ or DBH^NTS^ neurons induced action potentials (Fig. 5c,d) demonstrating that both inputs can efficiently activate CGRP^PBN^ neurons. Indeed, 2- to 5-ms pulses of blue light elicited excitatory currents in CGRP^PBN^ neurons from both inputs with millisecond latency (range 4–6 ms) and sub-millisecond jitter (0.23±0.036 and 0.22±0.028 ms for CCK, and DBH inputs, respectively; Fig. 5e–g), both indicative of monosynaptic connections^30^,^31^. Synaptic currents (amplitude 53±10.1 and 21.6±4.5 pA for CCK, and DBH inputs, respectively, MP=−80 mV) from both inputs were completely abolished by inhibitors of AMPA and NMDA receptors (Fig. 5h–j); thus, revealing glutamate as a principle transmitter at these synapses. These results support previous observations that ∼80% of DBH^NTS^ neurons are glutamatergic and that the majority of NTS to PBN transmission is via excitatory, glutamatergic neurons^5^,^32^. Of 52 patch-clamped CGRP neurons from 17 *Cck*^*Cre/+*^*::Calca*^*Cre:GFP/+*^ mice and 14 *Dbh*^*Cre/+*^*::Calca*^*Cre:GFP/+*^ mice, 12 responded to photoactivation of either input, suggesting that only a subset of CGRP^PBN^ neurons receive innervations from CCK^NTS^ or DBH^NTS^ neurons. Altogether, these data demonstrate that appetite-suppressing CGRP^PBN^ neurons^7^ are directly activated by glutamatergic CCK^NTS^ and DBH^NTS^ neurons.

### NTS to PBN axon stimulation decreases food intake

**Figure 6:**
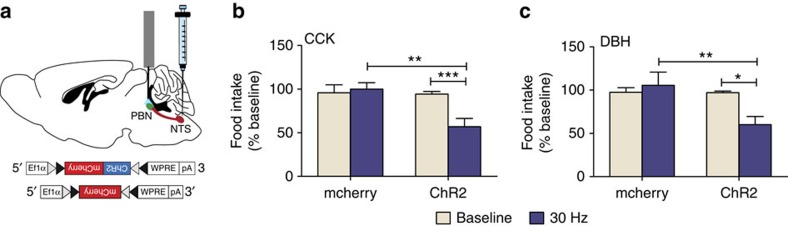
Activation of CCK^NTS^ and DBH^NTS^ axon terminals in the PBN suppresses food intake. (**a**) Cartoon of a sagittal mouse brain depicting site of viral injections for either AAV1-DIO-ChR2:mCherry or AAV1-DIO-mCherry into the NTS and placement of fibre optic cannula for light administration in the PBN. (**b**,**c**) Photoactivation of CCK^NTS^ (**b**) or DBH^NTS^ (**c**) axon terminals in the PBN decreased food intake when measured 2 h after lights out compared to baseline, non-stimulated food intake, or food intake by stimulating mCherry-only controls. Data (*n*=6 per group) represent mean±s.e.m., *P<0.05 **P<0.01, ***P<0.001 (two-way repeated measures ANOVA and bonferroni *post hoc* comparison).

Because CCK^NTS^ and DBH^NTS^ neurons send axonal projections to several brain regions, it was important to determine whether the projection to the PBN could be responsible for the suppression of food intake. *Cck*^*Cre/*+^ or *Dbh*^*Cre/+*^ mice were injected with AAV1-DIO-ChR2:mCherry or the control AAV-DIO-mCherry virus and fibre optic ferrules were placed above the lateral PBN (Fig. 6a). At least 3 weeks after viral injection, the mice were connected to a blue laser and either exposed to no stimulation (light off) or PBN terminals were photoactivated (30 Hz, 10 ms pulses, 1 s stimulation every 5 s) for 2.5 h starting 30 min before lights out. In previous studies, 30 Hz stimulation of CGRP^PBN^ neurons expressing ChR2 was sufficient to produce significant effects on feeding^7^,^33^ and induce expression of Fos^7^. In both *Cck*^*Cre/*+^ and *Dbh*^*Cre/+*^ mice, a significant decrease in food consumption was observed by both groups of ChR2-expressing mice during fibre stimulation within the PBN, when compared with their non-stimulated baseline intake or when compared with controls expressing only mCherry (Fig. 6b,c).

Photostimulation of ChR2-expressing terminals within the lateral PBN inhibited feeding; however, we cannot rule out the occurrence of antidromic action potentials activating cell bodies of the NTS neurons, which may then activate axon collaterals projecting to other brain regions. Indirect evidence suggests this is unlikely since low levels of Fos immunoreactivity were observed in NTS sections following 2 h of PBN stimulation in either *Cck*^*Cre/*+^ or *Dbh*^*Cre/+*^ mice expressing ChR2 (average per slice: 9.9±1.8 and 16.9±4.4 for *Cck*^*Cre/*+^ and *Dbh*^*Cre/+*^ mice, respectively, *n*=3 sections each from three animals per group); see Fig. 4 for background levels of Fos expression and the effects of direct activation of these NTS neurons.

## Discussion

Our study extends the neural network for anorexia by providing new insights into specific neuronal circuitry between the NTS and the PBN that suppresses appetite. Using electrophysiology coupled with optogenetics, we demonstrate direct excitatory connections from both CCK^NTS^ and DBH^NTS^ neurons onto CGRP^PBN^ neurons that have been shown to promote anorexia^7^. *In vitro*, these connections were capable of inducing action potentials in downstream CGRP^PBN^ neurons. Using chemogenetic (DREADD) and optogenetic (ChR2) approaches to selectively activate either CCK^NTS^ or DBH^NTS^ neurons or their terminals within the PBN *in vivo*, we establish that activation of either NTS population can decrease food intake. Because both populations of NTS neurons are activated by consumption of a large meal, our combined results suggest that they relay satiation signals from the gut to forebrain via CGRP^PBN^ neurons.

Activation of either CCK^NTS^ or DBH^NTS^ neurons with hM3Dq and CNO decreased feeding and induced Fos expression in CGRP^PBN^ neurons as well as neurons in the CeA and BNST that are known to be downstream targets of CGRP^PBN^ neurons^7^. Inactivation of CGRP^PBN^ neurons with tetanus toxin mitigates the anorexigenic responses to i.p. injections of CCK, the glucagon-like peptide-1 (GLP-1) agonist exendin 4, or leptin^26^. Altogether, these observations provide compelling evidence that the CCK^NTS^- and DBH^NTS^-to-CGRP^PBN^ pathways are among those that relay viscerosensory signals and promote satiation. There may be cross talk between NTS neuron populations that are activated by visceral input because hM3Dq-mediated activation of CCK^NTS^ neurons induces Fos not only in CCK neurons but also in neighbouring neurons, including DBH^NTS^ neurons.

The robust inhibition of food intake we observed in hM3Dq-expressing animals lasted 4–6 h when giving CNO injections twice daily with little difference in 24-h food intake. A single injection of CNO has variable duration peaking between 5 and 50 min and lasting between 1 and 9 h (refs 34, 35). The mice apparently compensated for the decreased food intake that occurred during the first few hours after CNO injection by ingesting greater amounts of food once the effect of CNO subsided. Although 24-h food intake was not affected, activation of either CCK^NTS^ or DBH^NTS^ neurons induced a modest, but significant, decrease in body weight. In addition to processing viscerosensory signals from the gut and relaying them to forebrain nuclei to inhibit feeding, these NTS neurons presumably influence energy expenditure. Therefore, activating CCK^NTS^ or DBH^NTS^ neurons in response to a large meal signals energy availability and promotes energy expenditure possibly via connections to the sympathetic nervous system^36^,^37^.

We demonstrate an important role for two genetically distinct NTS neurons in appetite suppression and provide evidence for a direct and functional connection with the lateral PBN. However, there are additional neuronal populations in the NTS that send projections to the PBN^4^. Therefore, the CCK^NTS^ and DBH^NTS^ projections to the CGRP^PBN^ neurons likely represent a subset of functionally relevant NTS projections to the PBN that mediate food intake. The NTS is a highly heterogeneous region containing multiple cell types. Just as in our refeeding study, many of the NTS neurons activated by gut distention, satiety hormones or anorexigenic agents are molecularly undefined^38^,^39^,^40^,^41^. A few other NTS cell types have been shown to affect feeding behaviour. For example, acute activation of pro-opiomelanocortin (POMC)-expressing neurons in the NTS using CNO and hM3Dq expression inhibits baseline feeding^42^. Increasing meal size or doses of the satiety hormone CCK activates increasing numbers of Fos-positive GLP-1- and prolactin-releasing peptide (PrRP)-expressing neurons in the NTS, suggesting that they may also relay satiation signals^17^,^18^. The presence of ghrelin and leptin receptors on multiple neuron populations in the NTS also suggests a role for NTS signalling in control of food intake^8^,^43^,^44^. There is notable overlap of neuropeptide expression within the NTS. For example, some NTS neurons expressing GLP-1 also express CCK and *vice versa*^8^. The majority of DBH^NTS^ neurons also co-express PrRP^18^,^45^. GLP-1 neurons also co-express somatostatin and met-enkephalin, but they appear to be distinct from A2, DBH^NTS^ neurons^19^,^46^,^47^.

The rodent PBN relays a wide variety of sensory signals (including taste, temperature, pain and visceral malaise) to forebrain nuclei^2^,^3^,^6^,^7^. The CGRP^PBN^ neurons appear to mediate conditions that threaten homoeostasis, ranging from mild satiation in response to a large meal, to visceral malaise due to nausea or inflammation, to pain produced by heat or foot shock^7^,^33^,^48^,^49^. Only a quarter of CGRP neurons tested responded to photostimulation of either CCK^NTS^ or DBH^NTS^ inputs. While this is consistent with a functionally specialized sub-population of these neurons, their relatively small fraction can also be explained by either insufficient expression of ChR2 in terminals, variability of light intensity in the slice or reduction of dendrites and axons during slice preparation. These experimental constraints imply that the actual number of CGRP neurons receiving CCK and DBH inputs is likely to be higher. Robust photoactivation of all CGRP^PBN^ neurons promotes freezing behaviour^49^. Our optogenetic and chemogenetic stimulation paradigms involving only CCK^NTS^- and DBH^NTS^-targeted CGRP^PBN^ neurons did not induce freezing behaviour; likewise, chemogenetic activation did not produce anxiety as measured by the open-field test, suggesting that the NTS to PBN circuit examined here does not relay aversive signals.

NTS neurons communicate viscerosensory information to the PBN as one important pathway in appetite suppression; however, they also project axons to other brain regions that have been implicated in modulating food intake^9^,^50^,^51^,^52^. CCK^NTS^ and DBH^NTS^ neurons are known to project to brain regions where we observed Fos induction; consequently, signalling in those brain regions may also modulate feeding. Our data add to the growing recognition of multiple genetically identified neural circuits that influence feeding behaviour. An understanding of these circuits and their interactions will undoubtedly open up new avenues for therapeutic intervention for a number of metabolic and feeding disorders. While we cannot determine whether disruptions in CCK^NTS^ or DBH^NTS^ neurons specifically contribute to pathology in humans, it is abundantly clear that disruption of normal signalling of either CCK or noradrenergic systems as a whole can promote feeding-related disorders. For example, polymorphisms in either CCK, or its receptor (CCK-1R) or β2-adrenoceptor genes have been linked to obesity and type 2 diabetes in humans^53^,^54^,^55^,^56^,^57^,^58^. We speculate that mutations in genes expressed in the neural circuit that we describe here may contribute to maladaptive eating patterns.

## Methods

### Mice

All animals were heterozygous male mice group housed before stereotaxic surgery and maintained on a 12-h light/dark cycle with *ad libitum* access to rodent diet (Picolab, #5053) unless otherwise stated. For behavioural testing, 3- to 5-month-old animals were individually housed 1 week before habituation in Biodaq cages. Mouse lines used were *Dbh*^*IRES-Cre/+*^, with *IRES-Cre* targeted to a site just downstream of the termination codon in the last exon (provided by Dr. Steven Thomas at University of Pennsylvania), *Cck*^*IRES-Cre/+*^ (Jackson laboratory, *Cck*^TM1.1(cre)Zjh^/^J^)^59^; and *Calca*^*Cre:GFP*^ mice (generated by Dr. Richard Palmiter at the University of Washington)^7^; all mouse lines were backcrossed to C57BL/6 mice for >6 generations. For electrophysiology studies, either *Dbh*^*Cre/Cre*^ or *Cck*^*Cre/Cre*^ homozygous mice were bred with *Calca*^*Cre:GFP/Cre:GFP*^homozygous mice (Jackson laboratory *Calca*^tm1.1(cre/EGFP)Rpa^)^7^. This breeding paradigm yielded double knock-in mice that were heterozygous for each allele. All animal experiments were approved by the University of Washington Institutional Animal Care and Use Committee (IACUC).

### Virus production

Cre-dependent pAAV-Ef1α-DIO-YFP, pAAV-Ef1α-DIO-mCherry and pAAV-Ef1α-DIO-ChR2:mCherry plasmids were provided by K. Deisseroth and Cre-dependent pAAV-hSyn-DIO-hM3Dq:mCherry DNA plasmid was provided by B. Roth. pAAV1-Ef1α-DIO-synaptophysin:mCherry plasmid was generated from pAAV-Ef1α-DIO-synaptophysin:GFP^7^ by replacing the GFP fragment with mCherry. Viruses were prepared in our laboratory using serotype 1 helper plasmid by transfecting HEK293 cells, purifying lysates through a series of sucrose and CsCl ultracentifugation steps and dialysis. Final pellets were suspended in 0.1 M phosphate-buffered saline (PBS) at a titre of approximately 2 × 10^9^ particles per μl.

### Stereotaxic surgery

Mice were anaesthetised with isoflurane vapour and secured in a stereotaxic frame (David Kopf Instruments). The head of the animal was tilted nose up by 10 degrees. After incision, the skull was exposed and two drill holes were created at the following coordinates from bregma (anteroposterior −6.38 mm, medial lateral: ±0.4 mm). A 5 μl Hamilton syringe with a 32-gauge needle was then inserted into each drill hole and lowered down −7.1 mm from bregma. Virus (300 nl) was injected at a rate of 150 nl min^-1^. After 5 min, the needle was retracted and the incision was closed with sterile suture. In mice used for *in vivo* optogenetic experiments two fibre optic cannulae (Doric Lenses) were implanted above the lateral PBN (anteroposterior:−5.1 mm, medial lateral: ±1.7 mm, dorsal ventral: 3.0 mm) and affixed to the skull using Metabond (Parkell) and dental cement.

### Slice optogenetics and electrophysiology

After allowing at least 2 weeks after surgery for viral expression, 8- to 20-week-old male mice were used for *in vitro* slice electrophysiology recordings 3–4 h after the start of the light cycle. Because stress-released hormones can modify neuronal networks and thus affect our observations, we handled mice with special precautions by deeply anaesthetizing them with isoflurane in their housing room before decapitation. Brains were quickly removed into ice-cold sucrose cutting solution (in mM: 11 D-glucose, 234 sucrose, 2.5 KCl, 1.25 NaH_2_PO_4_, 5 MgCl_2_, 0.2 CaCl_2_, 26 NaHCO_3_) continuously aerated with carbogen gas mixture (5% CO_2_, 95% O_2_). Coronal 200–250-μm thick slices were cut from NTS and PBN regions by a Leica TV1000S vibratome in ice-cold cutting solution and transferred for recovery to recording artificial cerebrospinal fluid (ACSF, in mM: NaCl 115, KCl 3, CaCl_2_ 2, MgCl_2_ 1, NaH_2_PO_4_ 1, NaHCO_3_ 25 and D-glucose 11; osm 295, pH 7.4 when aerated with carbogen) for 30 min at 33 °C. They were kept in the same carbogen-aerated solution at room temperature for at least 1 h before recordings. The recording chamber was perfused with carbogenated 33 °C ACSF at a rate of 1.5–2 ml min^−1^. CCK^NTS^ or DBH^NTS^ neurons and axons were identified by mCherry expression and CGRP neurons were identified by nuclear GFP expression using a Nikon ECLIPSE FN1 microscope, NIR Apo 40 × /0.8N.A. objective, Photometrics CoolSNAP ES2 camera. Because GFP and ChR2 have overlapping excitation spectra, GFP imaging can also result in unwanted ChR2 activation and transmitter release. To avoid this, we imaged GFP under conditions of globally inhibited synaptic transmission in ACSF with a high Mg^2+^/Ca^2+^ ratio and low Ca^2+^ (5 mM Mg^2+^and 0.1 mM Ca^2+^). Visually-guided patching was performed with the oblique LED illumination technique^60^,^61^ and IR-LED emitting 850-nm light positioned at 23–25^o^ near the meniscus. Patch pipettes were of 4–5 MΩ resistance when filled with intracellular solution (in mM: 100 Cs-methanosulfonate, 25 CsCl, 2 MgCl_2_, 10 HEPES, 0.4 EGTA, 4 ATP, 0.4 GTP, 10 phosphocreatine, mosm 295, pH 7.3). Resting membrane potential was measured immediately after forming whole-cell patch corresponding to a holding potential of 0 and corrected for liquid junction potential. Cell-input resistance was calculated based on steady-state holding current required for hyperpolarizing 5 mV/200 ms step from the resting membrane potential and recordings were rejected if the access resistance exceeded 20 MΩ. Synaptic currents were evoked by optical activation of ChR2 with 2- to 5-ms pulses of blue light (up to 3 mW optic power) either from a 473 nm laser (LaserGLOW), or an optic fibre positioned over the slice or from a high power 460 nm LED (UHP-LED, Prizmatix) and microscope objective. The same energy light pulses did not elicit any activity in CGRP neurons from mice not expressing ChR2, thus excluding a possibility of non-specific, heat-evoked responses under our conditions. Synaptic latency was measured as the time between onset of blue-light pulse and the onset of synaptic current. Synaptic jitter was calculated as a standard deviation in variation of synaptic latency over multiple trials (25–50) in a particular neuron. Currents were elicited at 10-s intervals between successive trials, recorded in 2–5 kHz bandwidth (A-M System patch-clamp amplifier), digitized at 20 kHz (Digidata 1440A, Molecular Devices) and acquired by pCLAMP10 software (Molecular Devices).

To test the effect of CNO on hM3Dq and mCherry-expressing cells, CNO was applied via superfusion in the presence of 20 μM CNQX, 100 μM D-AP5 and 100 μM picrotoxin. A glass tube (400 μm diameter) filled with extracellular ACSF plus inhibitors and 3 μM CNO was positioned over a slice just before superfusion and CNO was delivered to the slice by gravity for ∼30–40 s. Spiking was recorded in cell-attached configuration to be minimally invasive and preserve G-protein signal-transduction. The recording patch-pipette was filled with extracellular ACSF and spikes were loose-patch recorded at 0 holding current. The effect of CNO was measured for 30 s as the fold change in spiking rate was relative to the basal frequency before CNO. When indicated, AMPA and NMDA glutamate receptors were inhibited by a cocktail of CNQX (20 μM, Tocris) and D-AP5 (100 μM, Tocris) added to ACSF, and GABAA receptors were inhibited by picrotoxin (100 μM, Tocris).

### Food intake measurements

For non-food-restricted intake measurements, animals were individually housed in BioDAQ cages (Research Diets, Inc.) to continuously measure food intake. After 1 week habituation to the cages and food (Research Diets, Inc. D12450B), animals were injected with saline for 3 days before being injected with CNO (0.8 mg per kg body weight, i.p., Tocris) twice daily for 4 days. The two injections were given 30 min before lights out and 4 h after the onset of the light cycle. Daily body weight was measured for all animals 30 min before lights out.

For the food-restriction assay, animals were weighed daily and the amount of food was adjusted to bring animals to 85–90% of their pre-restriction body weight. Once animals were at 85–90% body weight, they were also given 0.3 g of the test diet (Bio-Serve dustless pellets, F0071) for 3 days to prevent neophobia during testing. This diet was chosen over normal chow as it allows for more accurate measurements. One day before testing, animals were injected with saline and placed into a new clean cage for 1 h. On test day 1 during the light cycle, animals were randomly given either saline or CNO (0.8 mg kg^−1^, i.p.) and placed into a clean cage 45 min before given access to 2 g of the test diet for 1 h after which food intake was measured and animals were returned to their home cages. On test day 2, the same procedure was repeated except that animals previously tested with CNO were given saline and *vice versa*.

For the fasting refeeding experiments, mice were fasted for 18 h. Mice in the refed group were given normal chow for 2 h (controls had no food). Animals were perfused at the end of the 2-h feeding period and food intake was measured.

### *In vivo* optogenetics

All animals were injected with both ChR2 and hM3Dq viruses. Animals were pre-screened for the ability of CNO to decrease baseline food intake greater than 40% at 2 and 4 h after lights out. Prescreening was performed to insure correct viral targeting to the NTS before optogenetic experiments were performed; approximately 60% of mice passed this screen. Animals were next acclimated to the fibre optic cables and feeding paradigm on the night before testing. On subsequent nights, animals were attached to fibre optic cables and all food was removed from the cage 2 h before lights out. At lights out, food was returned and measured again 2 h later. For baseline feeding (2 days) no stimulation occurred. 2-h non-stimulated baseline intake was not different between groups (Control group: 0.7±0.08 g; ChR2 group: 0.8±0.06 g). On the third day, blue light (473 nm) was delivered in 10-ms pulses at 30 Hz for 1 s every 5 s starting at 30 min before lights out and ending when food consumption was measured 2.5 h later.

### Open-field testing

Open-field testing was performed after mice had at least 5 days of *ad libitum* access to normal chow. One day before testing, animals were given an injection of saline equal to the volume of CNO to be given (0.8 mg kg^−1^ i.p.). The following day all mice were given CNO (0.8 mg kg^−1^ i.p.) 45 min before being placed in the outer circumference of a circular open-field arena and allowed to move freely for 10 min. The open field is a circular polypropylene container 42-cm in diameter with 36-cm high walls. The sessions were captured digitally and the amount of time animals spent in the centre of the field (denoted as a virtual circle 14.5 cm in diameter in the centre of the field) as well as velocity and distance travelled were measured using Ethovision software (Noldus).

### Immunohistochemistry

Three hours after CNO injections (0.8 mg kg^−1^) mice were anaesthetised with pentobarbital and perfused transcardially with PBS followed by 4% paraformaldehyde in PBS. Brains were extracted, post-fixed overnight and protected in 30% sucrose dissolved in PBS for cryosectioning (30-μm sections). Sections were collected in cold PBS.

Brain sections were permeabilized with 0.2% Triton X-100 (PBST) for 30 min and treated with a blocking solution containing PBST and 3% normal donkey serum (Jackson Immunoresearch) for 1 h at room temperature. Sections were incubated overnight at 4 °C in block solution containing goat polyclonal Fos antibody (1:500; Santa Cruz Biotech, sc-52) and either rabbit polyclonal DsRed antibody (1:1,000; Clontech, #632496) rabbit polyclonal tyrosine hydroxylase antibody (1:1,000; Millipore, AB152) or mouse monoclonal CGRP antibody (1:5,000; Abcam, ab81887). For fasting refeeding experiments, Chicken GFP antibody (1:10,000 Abcam, ab13970) was also used in the same sections. To visualize nuclei of CGRP neurons in the PBN of *Calca*^*CRE:GFP/+*^ mice, a rabbit polyclonal green fluorescent protein antibody (1:1,000; Life Technologies, A11122) was used. After primary antibody incubation and washes in PBS, sections were incubated in Cy5-cojugated, donkey anti-goat IgG, Alexa Fluor 594 donkey anti-rabbit IgG, and/or either Alexa Fluor 488 donkey anti-rabbit IgG or Alexa Fluor 488 donkey anti-chicken IgG (1:500; Jackson Immunoresearch) in block solution for 2 h at room temperature. Finally, sections were washed in PBS, mounted onto glass slides, and coverslipped with DAPI Fluromount-G (Southern Biotech, 0100-290) before image acquisition.

Quantification of Fos was performed bilaterally on every 6th section in the following areas: in the NTS from bregma −6.96 to −7.9 mm; PBN from bregma −4.9 to −5.45 mm (four sections from each area per mouse); amygdala from bregma −1.2 to −1.75 mm (approximately three sections per mouse) and on every third section in the BNST from bregma −0.2 to +0.1 mm (three sections per mouse). Images were collected on either a Nikon upright epifluorescent microscope with a QImaging Camera or a laser-scanning Olympus FV1200 confocal microscope. For co-localization, z-series images were taken and counts were performed from collapsed images generated in FV−10 Ver. 4.0a viewer.

### Statistics

Data were analysed using Prism 5.0 (GraphPad Software). Two animals were excluded from data analysis because post hoc histological analysis revealed no viral transduction as indicated by mCherry fluorescence. One animal was excluded from data analysis because post hoc histological analysis revealed fibre optic cannulae placement was anterior to the PBN. Figures were prepared using Photoshop and Illustrator CS5 (Adobe Systems).

### Data availability

Detailed statistics and data that support the findings of this study are available from the corresponding author on request.

## Additional information

**How to cite this article:** Roman, C. W. *et al.* Genetically and functionally defined NTS to PBN brain circuits mediating anorexia. *Nat. Commun.* 7:11905 doi: 10.1038/ncomms11905 (2016).

## Supplementary Material

Supplementary Information Supplementary Figures 1-6
