# Supplementary material for: Genetically and functionally defined NTS to PBN brain circuits mediating anorexia
Source: Nat Commun. 2016 Jun 15;7:11905. doi: 10.1038/ncomms11905 (PMC4912612; doi:10.1038/ncomms11905)
Supplement: Supplementary Information — Supplementary Figures 1-6 [file ncomms11905-s1.pdf]

Supplementary Figure 1

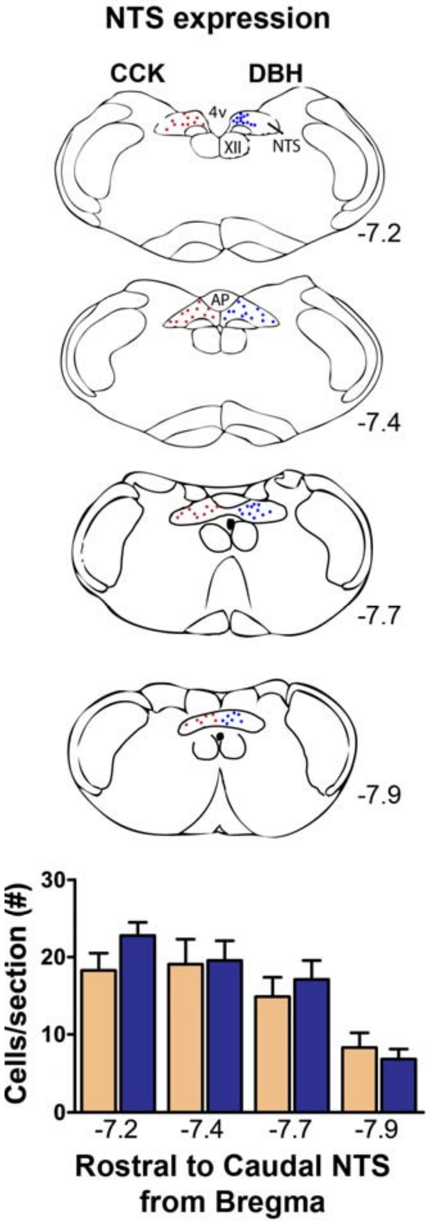

Supplementary Figure 1. Quantification of CCK- and DBH-expressing neurons throughout the caudal NTS. Coronal cartoon images representing the approximate number and spatial distribution for both CCK (left, red dots) and DBH (right, blue dots) neurons within 4 separate rostro-caudal levels of the NTS. Numbers to the right of sections denote approximate location as mm from bregma. Graph shows quantification of CCK and DBH expression across same NTS levels from a group of 17 mice (average of 3 sections per mouse).

Supplementary Figure 2

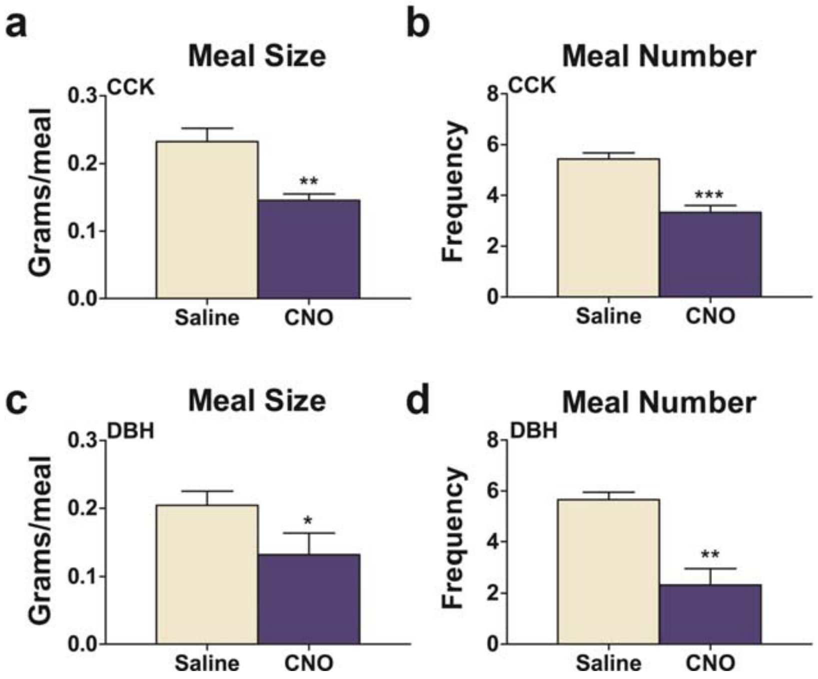

Supplementary Figure 2. Decreased meal size and number contribute to decreased feeding following activation of CCK^{NTS} and DBH^{NTS} neurons. a,c Chemogenetic activation of CCK^{NTS} (**a**) or DBH^{NTS} (**c**) neurons decreases average meal size during the first 4 h after lights out compared to average meal size following saline injection. **b,d,** Activation of CCK^{NTS} (**b**) or DBH^{NTS} (**d**) neurons decreases average number of meals during the first 4 h after lights out compared to saline injection. Meals were counted as >60 mg of food consumed with an inter-meal interval of >5 min. Data represent mean (n = 9 CCK, 6 DBH) ± s.e.m. (paired *t*-test). * $p < 0.05$, ** $p < 0.01$, *** $p < 0.001$.

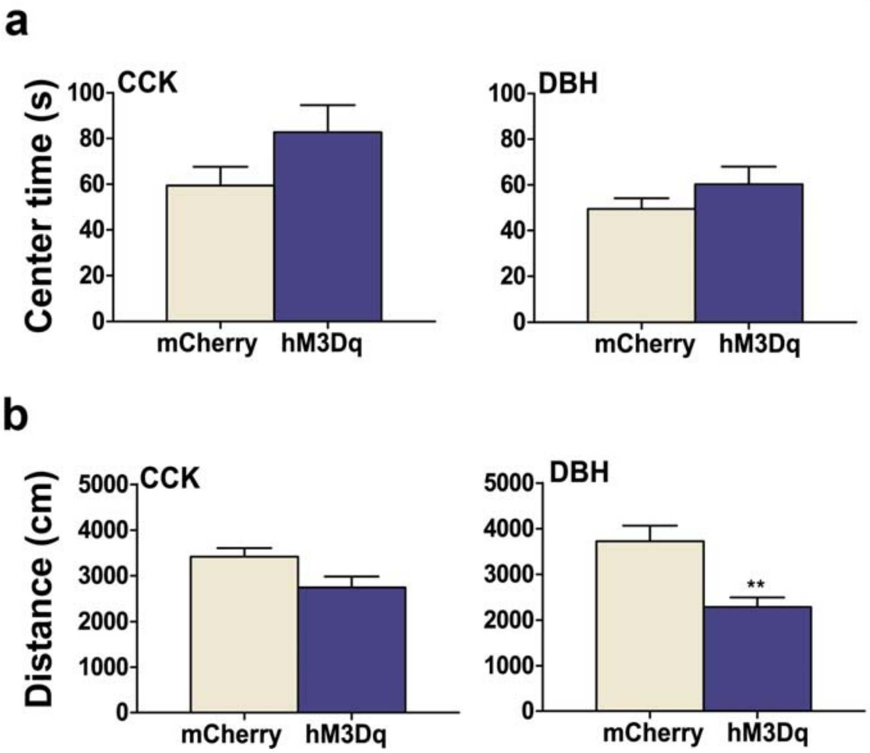

Supplementary Figure 3. Open-field test shows no changes in anxiety-like behavior following activation of CCK^{NTS} or DBH^{NTS} neurons. a, No significant differences were observed in time spent in the center of an open field arena 45 min after CNO injection between mCherry and hM3Dq groups for both $Cck^{Cre/+}$ and $Dbh^{Cre/+}$ mice. **b,** Distance traveled in the open-field was not different between groups for $Cck^{Cre/+}$ mice (left panel) but was significantly less in $Dbh^{Cre/+}$ mice expressing the hM3Dq receptor compared to mCherry controls. Data represent mean (CCK: n = 6 mCherry, 9 hM3Dq, DBH: n = 6 per group) ± s.e.m. (unpaired *t*-test). ** $p < 0.01$

Supplementary Figure 4

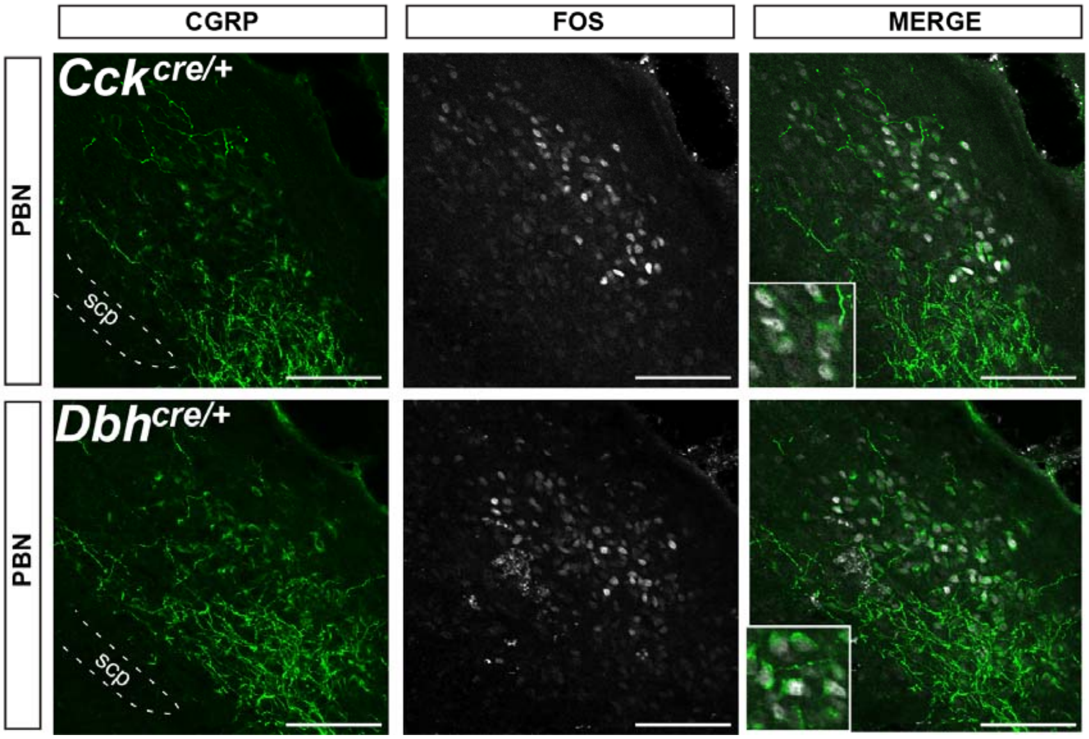

Supplementary Figure 4. CGRP^{PBN} neurons are activated following chemogenetic activation of CCK^{NTS} or DBH^{NTS} neurons. Representative histological images showing Fos- and CGRP-immunoreactivity in the PBN after chemogenetic activation of CCK^{NTS} (top panels) or DBH^{NTS} neurons (bottom panels). scp, superior cerebellar peduncle. Scale bar, 50 µm.

Supplementary Figure 5

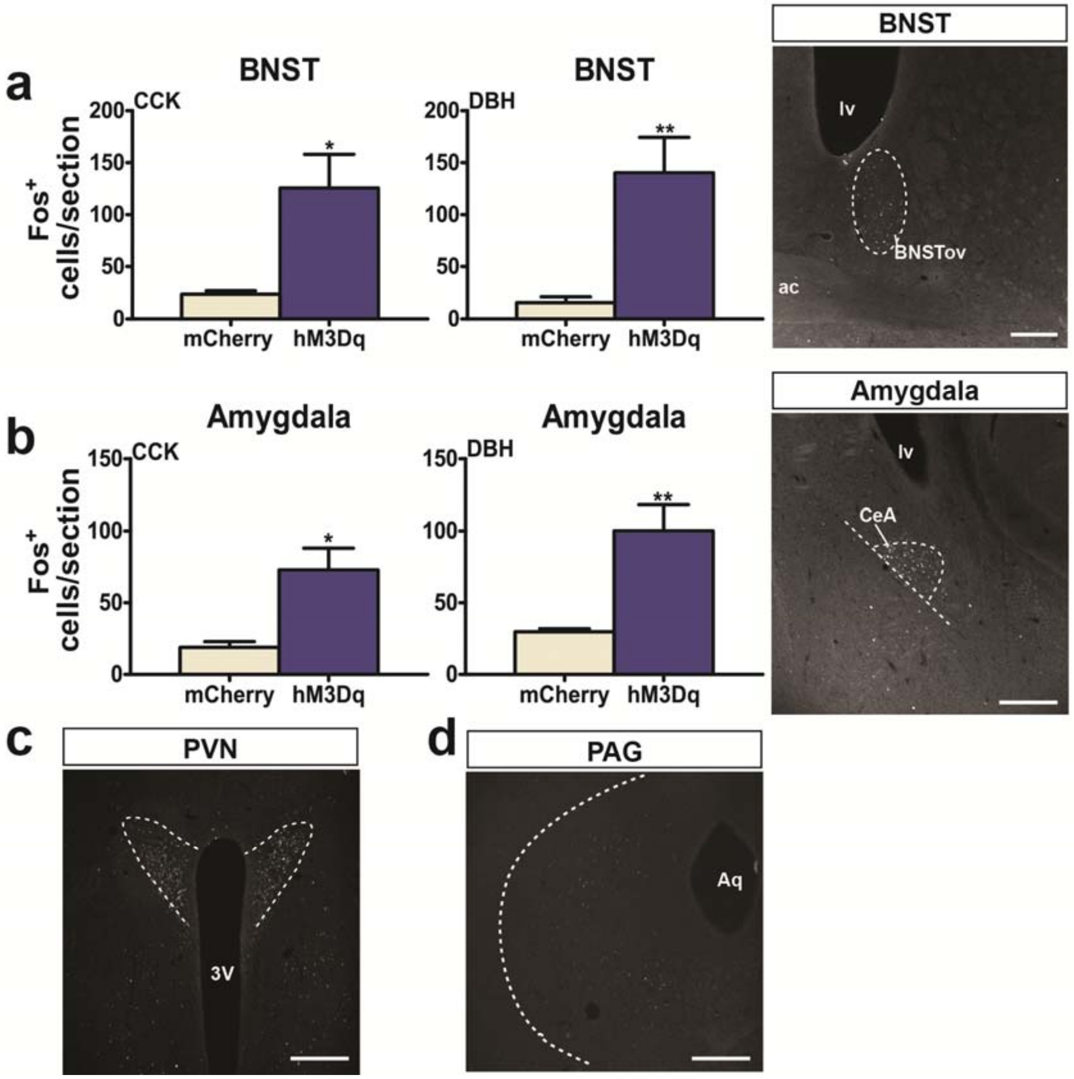

Supplementary Figure 5. Fos expression following activation of CCK^{NTS} and DBH^{NTS} neurons. a, Average number of Fos-positive cells per BNST section between groups in *Cck* $^{Cre/+}$ (left panel) and *Dbh*$^{Cre/+}$ mice (middle panel) after CNO injection. Representative section showing Fos immunoreactivity in the BNST in an hM3Dq-expressing mouse after CNO injection (right panel). Dotted line highlights dense expression in the oval nucleus of the BNST (BNSTov). **b,** Average number of Fos-positive cells per amygdala section between groups in *Cck* $^{Cre/+}$ (left panel) and *Dbh*$^{Cre/+}$ mice (middle panel) following CNO injection. Representative section showing Fos immunoreactivity in the amygdala in an hM3Dq-expressing mouse after CNO injection. Dotted line highlights dense expression in the central nucleus of the amygdala (CeA) (right panel). **c,d,** Representative section showing Fos immunoreactivity in the paraventricular nucleus of the hypothalamus (PVN)(**c**) and in the periaqueductal gray (PAG)(**d)** in an hM3Dq-expressing mouse after CNO injection. 3V, 3rd

ventricle. Aq, cerebral aqueduct. Scale bars, 50 µm. Data represent mean (CCK: n = 5 mCherry, 8 hM3Dq, DBH: n = 6 per group) ± s.e.m. * $p < 0.05$, ** $p < 0.01$ (unpaired *t*-test).

Supplementary Figure 6

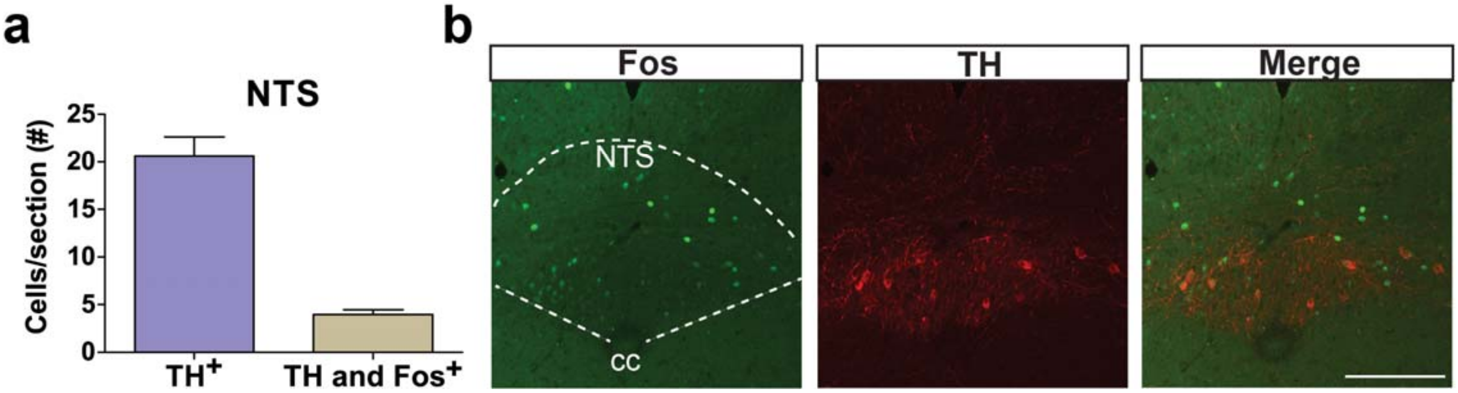

Supplementary Figure 6. Activation of CCK^{NTS} neurons stimulates local NTS neurons including noradrenergic cells. a, Average number of NTS cells per section immunopositive for TH, a marker for DBH-expressing, noradrenergic neurons compared with average number of cells per section expressing markers for both TH and Fos after chemogenetic activation of CCK^{NTS} neurons. Data represent mean (n = 38 sections from 6 animals) ± s.e.m. **b,** Representative histological section through the caudal NTS showing TH immunopositive cells, and their processes, in red and Fos positive cells (nuclei) in green. Scale bar, 100 µm.
